# A *HS6ST2* gene variant associated with X‐linked intellectual disability and severe myopia in two male twins

**DOI:** 10.1111/cge.13485

**Published:** 2018-12-26

**Authors:** Leda Paganini, Loubna A. Hadi, Massimiliano Chetta, Davide Rovina, Laura Fontana, Patrizia Colapietro, Eleonora Bonaparte, Lidia Pezzani, Paola Marchisio, Silvia M. Tabano, Jole Costanza, Silvia M. Sirchia, Laura Riboni, Donatella Milani, Monica Miozzo

**Affiliations:** ^1^ Division of pathology, Research Laboratory Unit, Fondazione IRCCS Ca' Granda Ospedale Maggiore Policlinico Milan Italy; ^2^ Medical Genetics, Department of Pathophysiology and Transplantation Università degli Studi di Milano Milan Italy; ^3^ Department of Medical Biotechnology and Translational Medicine, LITA‐Segrate Università degli Studi di Milano Milan Italy; ^4^ U.O.C. Genetica Medica e di Laboratorio, A.O.R.N. Cardarelli Naples Italy; ^5^ Medical Genetics, Department of Health Sciences Università degli Studi di Milano Milan Italy; ^6^ Pediatric Highly Intensive Care Unit Fondazione IRCCS Ca' Granda Ospedale Maggiore Policlinico Milan Italy

**Keywords:** *HS6ST2*, intellectual disability (ID), syndromic myopia, whole exome sequencing (WES)

## Abstract

X‐linked intellectual disability (XLID) refers to a clinically and genetically heterogeneous neurodevelopmental disorder, in which males are more heavily affected than females. Among the syndromic forms of XLID, identified by additional clinical signs as part of the disease spectrum, the association between XLID and severe myopia has been poorly characterized. We used whole exome sequencing (WES) to study two Italian male twins presenting impaired intellectual function and adaptive behavior, in association with severe myopia and mild facial dysmorphisms. WES analysis detected the novel, maternally inherited, mutation c.916G > C (G306R) in the X‐linked *heparan sulfate 6‐O‐sulfotransferase 2 (HS6ST2)* gene. HS6ST2 transfers sulfate from adenosine 3′‐phosphate, 5′‐phosphosulfate to the sixth position of the N‐sulphoglucosamine residue in heparan sulfate (HS) proteoglycans. Low HS sulfation levels are associated with defective optic disc and stalk morphogenesis during mammalian visual system development. The c.916G>C variant affects the HS6ST2 substrate binding site, and its effect was considered “deleterious” by *in‐silico* tools. An in‐vitro enzymatic assay showed that the HS6ST2 mutant isoform had significantly reduced sulphotransferase activity. Taken together, the results suggest that mutant HS6ST2 is possibly involved in the development of myopia and cognitive impairment, characteristics of the probands reported here.

**Abbreviations**
BMP: bone morphogenetic protein
BWB syndrome: Brooks Wisniewski Brown syndrome
DTT: dithiothreitol
FGF: fibroblast growth factor
GlcNS: N‐sulfoglucosamine
HS: heparan sulfate
HS6ST2: heparan sulfate 6‐O‐sulfotransferase 2
HSPG: heparan sulfate proteoglycan
ID: intellectual disability
NGS: next‐generation sequencing
PAPS: adenosine 3′‐phosphate, 5′‐phosphosulfate
RA: retinoic acid
SHH: sonic hedgehog
WES: whole exome sequencing
XLID: X‐linked ID

## 1 INTRODUCTION

Intellectual disability (ID) is defined as a neurodevelopmental disorder with onset before 18 years of age, characterized by IQ ≤ 70 and deficit in at least two adaptive behaviors (eg, communication, self‐care).^1^ ID occurs in approximately 1% to 3% of the population^2^ and has a variety of environmental and genetic causes, which are frequently present in combination with one another.^3^ Mild ID is typically associated with environmental features, while genetic factors have a major influence in moderate, severe, and profound ID^4^; however, the identification of factors contributing to ID etiology is complicated by the extreme heterogeneity of this condition. Numeric and structural chromosomal aberrations, as well as variants in at least 800 protein‐coding genes,^3^ explain approximately half of ID diagnoses, while the etiological factors underlying the remaining cases are unknown. Mutations in genes influencing ID can give rise to both syndromic and non‐syndromic forms of the condition. Syndromic ID includes a highly heterogeneous group of phenotypes where ID occurs together with a constellation of other features.^5^ ID gene density is particularly high on the X chromosome, with more than 10% of all ID‐related protein‐coding genes mapping to the X, establishing the condition referred to as X‐linked ID (XLID) that predominantly occurs in males^6^; overall, 30% more males are affected than females.^7^

Here, we report two monozygotic male twins of a triplet pregnancy with clinical diagnosis of syndromic ID combined with severe myopia and mild dysmorphic features.

High myopia is a prominent refractive error. Gupta et al. reviewed the candidate genes related with inherited myopia and reported only two forms of non‐syndromic condition, one involving *ZNF644* and the other related to *SCO2* gene.^8^ High myopia and severe ID, presenting as isolated manifestations without other relevant dysmorphic or malformative features, have never been previously associated with a known clinical condition. Conversely, they have been identified in many syndromes, such as Donnai‐Barrow or Cohen syndromes, as part of a broad range of clinical signs affecting many parts of the body.

Genetic investigation of the triplets and their immediate family revealed a mutation in the *heparan sulfate 6‐O‐sulfotransferase 2* (*HS6ST2*) gene on the X chromosome, predicted to detrimentally influence protein function. in vitro assays confirmed the deleterious effect of the mutation on HS6ST2 enzyme activity. Hence, mutations in *HS6ST2* represent a potential new cause of syndromic XLID.

## 2 MATERIALS AND METHODS

### 2.1 Study aim, design, and setting

The aim of this study was to screen for the genetic basis of the phenotype of two identical twins presenting at our hospital with ID and severe myopia. Following clinical examination, whole exome sequencing (WES) and functional analysis of the potential causal mutation were performed. The study setting was the Fondazione IRCCS Ca' Granda Ospedale Maggiore Policlinico, Milano, Italy. Appropriate informed consent for WES analysis for research purposes was obtained from all family members.

Parental approval was also obtained for the inclusion of the genetic data of all family members in a scientific publication.

### 2.2 Whole exome sequencing

Genomic DNA was extracted from peripheral blood samples of all family members using a QIAamp DNA mini kit (Qiagen, Hilden, Germany), according to the manufacturer's instructions. DNA was quantified using a NanoDrop spectrophotometer (Thermo Fisher Scientific, Waltham, MA). A SureSelect Clinical Research Exome V5 kit (Agilent, Santa Clara, CA) was used to perform next‐generation sequencing (NGS) analyses of DNA samples (2 μg). Libraries were analyzed by paired‐end NGS (NextSeq 550; Illumina, San Diego, CA).

Sequences were aligned to the human genome reference (GRCh37/hg19). WES data were analyzed using Illumina VariantStudio data analysis software and annotated and filtered with wANNOVAR (http://wannovar.wglab.org).

Autosomal and X‐linked recessive transmissions were first investigated as potential inheritance patterns. De novo variants were also considered. Accordingly, we set the variant calling parameters as follows: PassFilter = ON; Quality>30; Read Deph>8; Alt Variant Freq>25. We first focused our analysis on consensus splice‐site changes, non‐synonymous variants, and insertions/deletions in exonic regions. We also assumed that the alternative allele should have a frequency lower than 0.01 (based on Exome Aggregation Consortium [ExAC] all frequency) or that it should be absent from the ExAC, 1000 Genomes, dbSNP132, COSMIC and ClinVar databases. Output files from VariantStudio are provided as Supporting Information Appendix S1, S2, and S3 (autosomal recessive, X‐linked recessive and de novo variants, respectively).

*HS6ST2* variant*,* identified in probands FII‐1 and FII‐2 and in their mother, was confirmed by Sanger sequencing using the following primers: Forward, 5′‐AGCAGCGGCTCCAGGGGAAG‐3′ and Reverse, 5′‐GTCAGGGTGCGCGCTAGGTC‐3′.

### 2.3 Bioinformatic analyses

In silico prediction of the functional effect of the c.916G>C mutation on the *HS6ST2* gene was performed using the “Mutation Taster” tool. At the protein level, the consequences of the Gly306Arg substitution on HS6ST2 tertiary structure were evaluated using Phyre2 software (Protein Homology‐fold Recognition Server; www.sbg.bio.ic.ac.uk/phyre2/). Wild‐type and mutated HS6ST2 3D structures were modelled.^9^ These analyses were implemented using Phyre investigator, a workbench analysis tool with additional features for investigation of models created using Phyre2. All *.pdb files generated using Phyre2 were loaded and visualized with ChemDraw (version 8; Cambridge Software; PerkinElmer, Inc., Waltham, Massachusetts).

### 2.4 *HS6ST2* site‐directed mutagenesis

The mammalian pReceiver‐M12 Ampr vector containing the full length *HS6ST2* cDNA isoform (NM_001077188.1, ENST00000521489.5) fused to an N‐terminal 3× Flag‐tag (tebu‐bio) was used as template to generate a *HS6ST2* c.916G>C mutant sequence, by site‐directed mutagenesis (QuikChange II Site‐Directed Mutagenesis Kit; Stratagene, San Diego, CA). Primers used to obtain the G>C nucleotide substitution were as follows: Forward, 5′‐GCCCTCCGT GGTGGAC**C**GCAAGCGCGACGCCAG‐3′ and Reverse, 5′‐CTGGCGTCGCGCTTGC**G**GGTGGACACGGAGGGC‐3′.

The plasmid expressing the Gly306Arg mutant was transformed into *Escherichia coli* strain DH5α for amplification. The presence of the c.916G>C transversion inside the plasmid was confirmed by Sanger sequencing using the following primers: Forward, 5′‐GCCCTCCGTGGTGGACCGCAAGCGCGACGCCAG‐3′ and Reverse, 5′‐CTGGCGTCGCGCTTGCGGTCCACCACGGAGGGC‐3′.

### 2.5 Immunoblot assay

Wild‐type and mutant (G306R) plasmids were transiently transfected into HEK293T (human embryonic kidney) cells cultured in Dulbecco's Modified Eagle's Medium (DMEM), using Lipofectamine 2000 (Invitrogen, Carlsbad, CA) according to the manufacturer's protocol. Total protein extracts (20 μg) from cell lysates were electrophoretically separated on 10% denaturing polyacrylamide gels and then transferred to Polyvinylidene difluoride (PVDF) membranes (Roche, Basilea, Switzerland). A high range SigmaMarker (Sigma‐Aldrich, Saint Louis, MO; molecular weight range, 36 000‐200 000 kDa) was used to characterize protein migration. Membranes were blocked in Tris‐buffered saline with 0.1% Tween 20 containing 5% non‐fat dried milk and reacted overnight at 4°C with the primary monoclonal antibody, anti‐FLAG mouse M2 clone (Sigma‐Aldrich) diluted 1:2000, and anti‐beta Actin antibody ab8226 (Abcam, Cambridge, UK), diluted 1:1000. After rinsing, filters were incubated with horseradish peroxidase‐conjugated goat anti‐mouse IgG secondary antibody (Santa Cruz), diluted 1:10000. Signals were developed using the enhanced chemiluminescent horseradish peroxidase (HRP) substrate Westar ETA C ultra (Cyanagen, Bologna, Italy), and specific protein bands were revealed using the G:BOX Chemi XT4 gel doc system (Syngene, Frederick, MD).

### 2.6 Assay of HS6ST2 activity

The enzymatic activity of HS6ST2 was assayed in cell extracts from either wild‐type or mutant (G306R) transiently transfected HEK293T cells, incubated in DMEM for 48 hours. The enzyme assay was performed according to Habuchi et al.,^10^ with minor modifications. To obtain cell extracts, cells were washed twice with cold phosphate‐buffered saline, harvested, and then resuspended in the following Buffer A: 10 mM Tris/HCl, pH 7.2, 0.15 M NaCl, 10 mM MgCl2, 2 mM CaCl2, 20% (w/v) glycerol, and 0.5% (w/v) Triton X‐100. After stirring for 1 hour at 4°C in a rotatory shaker, cell homogenates were centrifuged at 10000 × g for 30 minutes at 4°C. Supernatants (cell extracts) were carefully collected, and their protein content was determined,^11^ followed by assay of HS6ST2 activity.

The reaction mixture (50 μL, final volume) contained 50 mM imidazole‐HCl (pH 6.8), 75 μg/mL protamine chloride, 150 mM NaCl, 20 mM NaF, 500 μM HS (as glucuronic acid), 10‐30 μg cell extract protein, and 2 μM [35S] PAPS (2 Ci/mmol). When indicated, dithiothreitol (DTT) (2‐10 mM) was added. The reaction mixtures were incubated for 15‐30 minutes at 37°C, and then stopped by heating the tubes at 100°C for 1 minute. After cooling at 4°C, chondroitin sulfate A (50 nmol, as glucuronic acid) was added as a carrier to facilitate precipitation. The mixtures were then supplemented with four volumes of 95% ethanol/1.3% (w/v) potassium acetate/0.5 mM ethylenediaminetetraacetic acid (EDTA), mixed and maintained at −20°C for 30 minutes. [35S]‐labeled glycosaminoglycans were precipitated by centrifugation (10 000 × g for 10 minutes at 4°C), resuspended in water and separated from unreacted [35S] PAPS and inorganic sulfate using a Sephadex G‐25 superfine column (Amersham Bioscience, Little Chalfont Buckinghamshire, UK), following the manufacturer's instructions. The eluate, containing [35S]‐labeled polysaccharides, was collected by centrifugation and the radioactivity was measured by liquid scintillation using a beta‐counter (TRICARB 2100‐TR; Perkin Elmer Life Sciences, Boston, Massachusetts).

## 3 RESULTS

### 3.1 Probands

**Figure 1:**
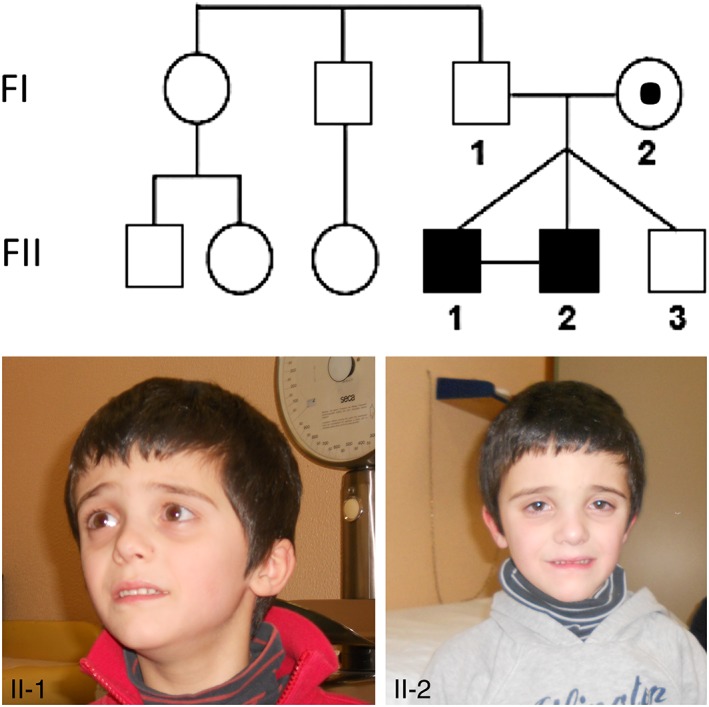
Pedigree of individuals carrying the HS6ST2 p.G306R (c.916G > C) variant in this study. Photographs of the two affected monozygotic twins (FII‐1 and FII‐2) are presented. Filled symbols represent symptomatic individuals, open symbols represent unaffected individuals, and the open symbol with a black dot represents an individual who carries the mutation. Monozygotic twins are recognizable from the connected symbol [Colour figure can be viewed at wileyonlinelibrary.com]

An Italian family composed of healthy non‐consanguineous parents (FI‐1 and FI‐2), two affected male twins (FII‐1 and FII‐2) and one healthy brother (FII‐3) (Figure 1) was investigated. The triplets were born at 30 weeks' gestation by emergency cesarean section, due to fetal distress. The pregnancy, resulting from in vitro fertilization, was dichorionic‐triamniotic and the twins were two identical (FII‐1 e FII‐2) and one brother (FII‐3). The probands came to our attention when they were 5 years old, after a diagnosis of severe psychomotor delay. Karyotype and comparative genomic hybridization (CGH) array results were normal, and they were negative for *FMR1* repeat expansion. The clinical features of the probands are detailed in Table 1. Both had neurodevelopmental impairment, classified as “severe” according to the Griffith scale (score = 34), and speech delay. They began walking at 2 years old and could speak only poorly at 5 years old. A routine growth checkup at 5 years old revealed weight in the 10th and height in the 25th percentiles. In the first year of life, they suffered from febrile seizures, with evidence of epileptiform electroencephalography (EEG) abnormalities, which presented as diffuse and irregular spikes. Brain magnetic resonance imaging scan showed mild lateral ventricular enlargement. Both the probands exhibited high myopia (−6 diopters, compared with −0.75 diopters for their healthy sibling), accompanied by chorioretinopathy. They had mild dysmorphic facial features (Figure 1), including triangular face, large forehead, deeply set and halonated eyes, straight and downward‐slanting eyebrows, thin lips with downturned corners, slight prognathism, pointed chin, and small low‐set and malrotated ears. At the last checkup, performed at the age of 10, the children presented with feeding difficulties and urinary incontinence. Myopia remained severe but stable, and epilepsy was no longer present.

**Table 1:** Clinical features of the two affected twins

Clinical signs	II‐1 proband	II‐2 proband
Bifrontal narrowness	+/−	+/−
Low‐set ears	+	+
Deeply set eyes	+	+
Myopia	+	+
Halonated eyes	+	+/−
Wide nose tip	+/−	+/−
Triangular face	+	+
Malar flatness	+	+
Thin upper lip	+	+/−
Neurodevelopmental delay	+	+
Febrile convulsions	+	+
Lateral ventricular enlargement	+	+

+ indicates the presence of the sign; +/− indicates that the feature is present in a mild form.

Biochemical analyses, performed at 5 years old, revealed an elevated lactate/pyruvate ratio in FII‐1 patient (34.11; reference value: < 25), and FII‐2 patient had an increased blood lactic acid value (23 mg/dL; control range: 6.3‐18.9 mg/dL). Furthermore, alanine levels were elevated in both probands (values not available). Ranges of serum pyruvate and excreted urine organic acids were normal. At 10 years old, blood lactic acid values were only slightly increased in both patients (FII‐1:1.6 mM; FII‐2:1.4 mM; reference range: 0‐1.3 mM). Notably, the two affected twins had low glycemic scores (FII‐1:67 mg/dL; FII‐2:68 mg/dL; normal range: 70‐110 mg/dL).

Input of these clinical and genetic evidences into the Face2Gene next‐generation phenotyping application indicated a possible mild correlation with the phenotype of Brooks Wisniewski Brown (BWB) syndrome (OMIM: #300612).

### 3.2 Mutation analysis by NGS

Using WES analysis, we identified the hemizygous single‐nucleotide variant, c.916G>C (p.G306R), in the *HS6ST2* gene (RefSeq NM_001077188.1; NP_001070656.1) on chromosome Xq26.2. The enzyme encoded by *HS6ST2* is responsible for the transfer of sulfate from adenosine 3′‐phosphate, 5′‐phosphosulfate (PAPS) to the sixth position of the N‐sulfoglucosamine (GlcNS) residue in heparan sulfate (HS) proteoglycans (HSPGs). Two different splice isoforms of human *HS6ST2* have been identified and exhibit tissue‐specific localization: full length *HS6ST2* is expressed in the eyes and brain during embryonic development, and a short form of the gene, *hHS6ST‐2S*, encoding 40 fewer amino acids, has been detected in the ovary, placenta, and fetal kidney.^12^ Both isoforms catalyze the same sulfation reaction.^13^

The novel G>C transversion in *HS6ST2* identified here changes a GGC codon, encoding a non‐polar glycine residue, to a CGC, which encodes a positively charged arginine. The variant was present in the affected triplets (FII‐1 and FII‐2), carried by the healthy mother, and absent in both the father and the unaffected brother (FII‐3) (Figure 1). Sanger sequencing confirmed the findings of NGS and the segregation of the transversion in the family. The “Mutation Taster” in silico prediction tool classified the c.916G>C substitution as *deleterious*, with a score of 1.000 (values close to 1 indicate a high “security” of prediction). This substitution has never been reported in the main databases of nucleotide variants, including dbSNP, 1000 Genomes, and Exac (Appendix S1).

### 3.3 In silico 3D modeling of the HS6ST2‐G306R protein

**Figure 2:**
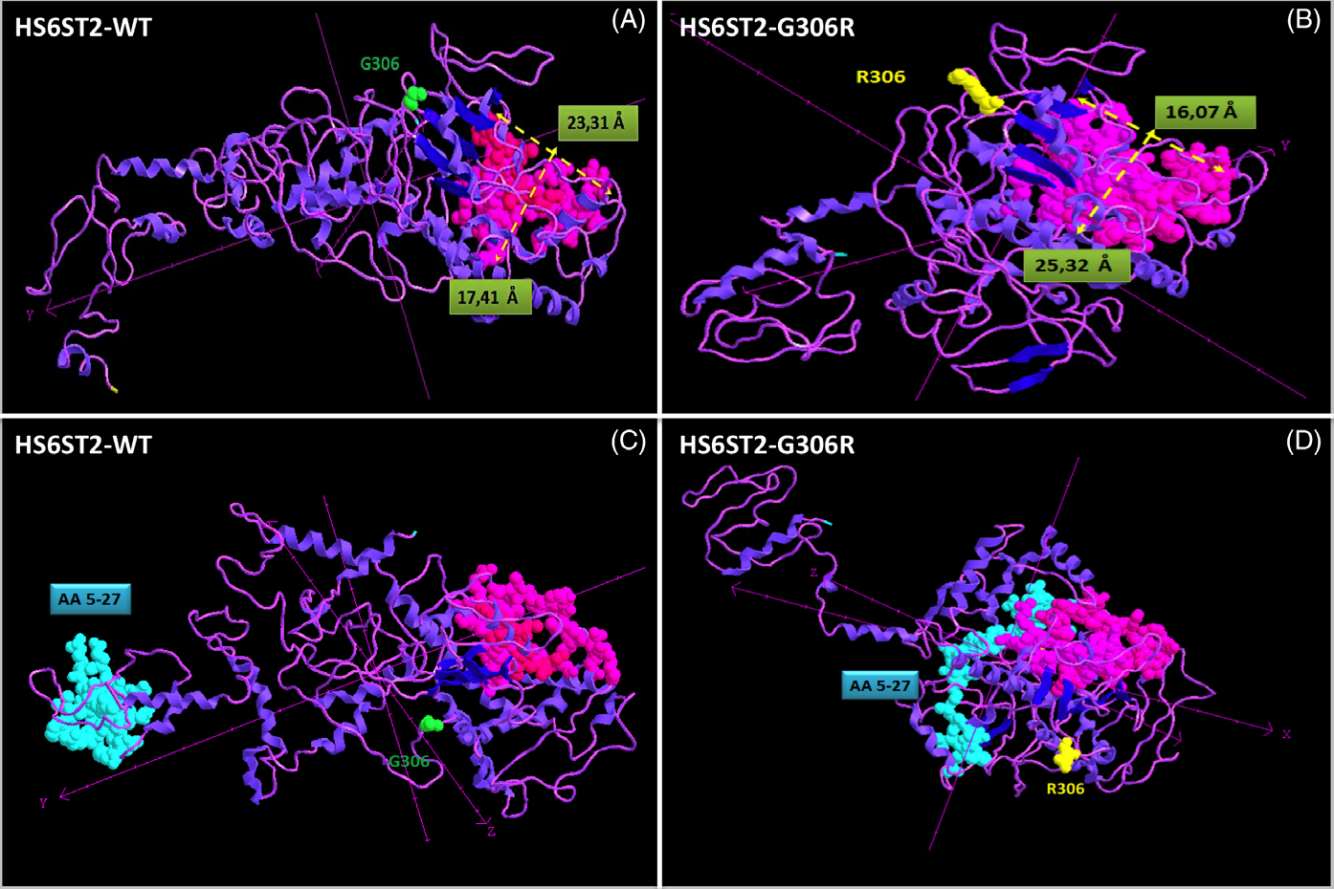
Wild‐type (A and C) and mutant (B and C) HS6ST2 3D structures, modeled using Phyre2 (protein homology‐fold recognition engine). In all panels, the amino acids forming the pocket surface are represented in light pink space‐filled mode, and the variant position (306) is indicated. A and B show pocket diameters (23.31 and 16.07 Å) and pocket depth values (17.41 and 25.32 Å) of the HS6ST2‐WT (A) and HS6ST2‐G306R (B) isoforms, respectively. C and D demonstrate the mislocation of the N‐terminal signal‐anchor domain (amino acids 5‐27), here highlighted in blue, in the WT (C) and mutant (D) HS6ST2 [Colour figure can be viewed at wileyonlinelibrary.com]

The G306R variant is located within the HS 6‐O‐sulfotransferase domain of the HS6ST2 protein, between the 5′‐phosphosulfate‐binding site and the 3′‐phosphate‐binding site (ENST00000521489.5). Three‐dimensional protein structures of wild‐type HS6ST2 and the G306R mutated variant were predicted with Phyre2 software, using the primary amino acid sequences as input (Figure 2). The G306R substitution was predicted to cause a significant change in the concave surface of the HS6ST2 protein, generating a deeper pocket with a smaller diameter (diameter, 16.07 Å; greatest depth, 25.32 Å; Figure 2, panel B) relative to the wild‐type structure (diameter, 23.31 Å; greatest depth, 17.41 Å; Figure 2, panel A). 3D conformation of the HS6ST2 6‐O‐sulfotransferase domain functions to maintain contact between the sulfate donor, PAPS, and position 6 of the acceptor GlcNS of HS. Changing the pocket surface, as well as the 3D structure, of the HS6ST2 active site could result in modification of its enzyme activity. Moreover, the G306R variant modifies protein conformation at the N‐terminus of HS6ST2, leading to a complete mis‐localization of the type II membrane protein signal‐anchor domain (amino acids 5‐27), which may lead to a failure of the protein to anchor to the Golgi apparatus membrane (Figure 2, panels C and D**;** amino acids corresponding to the N‐terminal domain are highlighted in light blue).

### 3.4 In vitro assay of HS6ST2‐G306R activity

**Figure 3:**
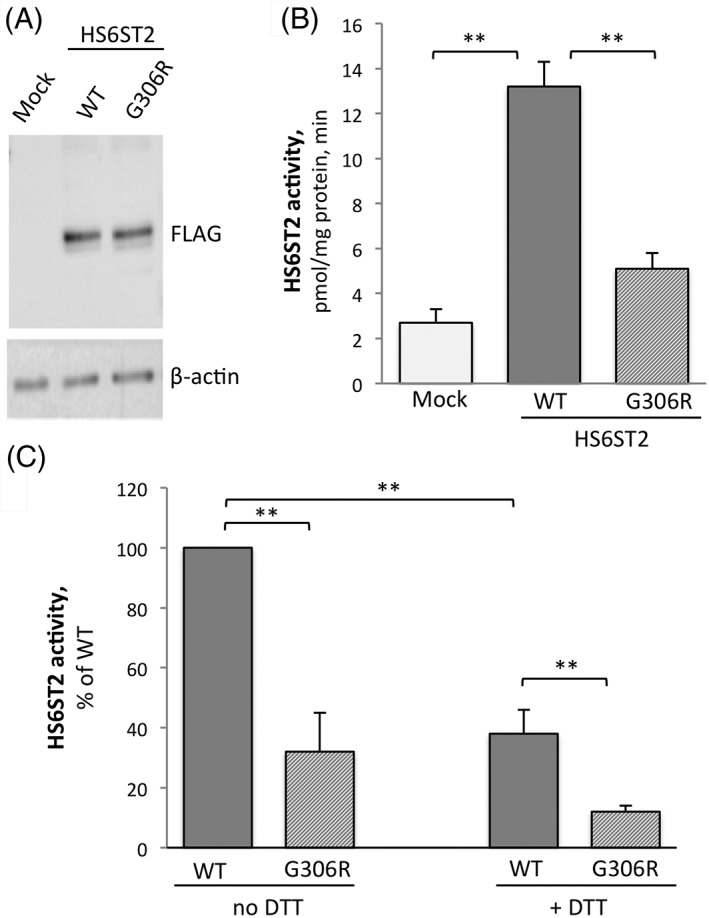
Expression and activity of HS6ST2 in mock, wild type and G306R mutant cells. HEK293 cells were transiently transfected with empty vector (mock), or with plasmids expressing wild‐type FLAG‐HS6ST2 (WT), or mutant FLAG‐G306R (G306R), and then subjected to immunoblot analysis (A), and the HS6ST2 assay (B and C). A, representative immunoblot image of HS6ST2 expression in mock, wild type and G306R mutant cells. β‐actin was used as loading control. B and C, HS6ST2 activity in the cell extracts of mock, WT and G306R cells. Enzyme activity was assayed as described in Materials and Methods in the absence (B) or presence (C) of 10 mM dithiothreitol (+ DTT). In all cases, the reaction rate was linear with enzyme protein and incubation time. Data are the mean ± SD of three individual assays. Statistical significance was assessed using two‐tailed Student's *t* test; **P < 0.01

To test whether G306R variant affects HS6ST2 enzyme expression and/or activity, HEK293 cells were transiently transfected with the empty vector (mock control) and with plasmids expressing wild‐type (HS6ST2‐WT) or G306R mutant (HS6ST2‐G306R) FLAG‐HS6ST2. We first analyzed the expression of recombinant wild‐type and mutant HS6ST2 proteins in HEK293 cells. Protein expression was undetectable in mock‐transfected cells but was evident in the HS6ST2‐WT and HS6ST2‐G306R transfected cells. As shown in Figure 3A, the expression levels of the wild‐type and mutant proteins were very similar.

Next, we examined HS6ST2 activity using HS as a substrate. In mock‐transfected HEK293 cells, HS6ST2 activity was 2.7 ± 20.6 pmol/mg protein, min. In HS6ST2‐WT transfected cells, 6‐O‐sulfotransferase activity was approximately 4‐fold higher at 13.2 ± 31.1 pmol/ mg protein, min, (*P* = 0.0087, two‐tailed *t* test), reflecting over‐expression of HS6ST2 from the transfection vector the enzyme activity increased approximately 4‐fold, to reach values of 13.2 ± 31.1 pmol/ mg protein, min, (*P* = 0.0087, two‐tailed *t* test). Conversely, in HS6ST2‐G306R transfected cells, 6‐O‐sulfotransferase activity (5.1 ± i0.7 pmol/min, mg; *P* = 0.0063, two‐tailed *t* test) was considerably lower than in HS6ST2‐WT transfected cells, demonstrating the importance of residue 306 for HS6ST2 enzymatic activity (Figure 3B). Because HS6ST (but not HS2ST) isoforms are inhibited by DTT 10, we next evaluated the effect of DTT on enzyme activity. As shown in Figure 3C, DTT caused an approximately 3‐fold decrease in both wild‐type and G306R enzyme activity, from 100% to 32% ± 13% (*P* = 0.0039, two‐tailed *t* test) and from 38% ± 8% to 12% ± 2% (*P* = 0.047, two‐tailed *t* test), respectively.

## 4 DISCUSSION

In this study, WES was performed to characterize genetically two affected monozygotic twins with an inconclusive clinical diagnosis. We detected a novel pathogenic variant of the X‐linked *HS6ST2* gene associated with a very unusual combination of syndromic ID and severe congenital myopia. The deleterious effect of the mutant enzyme was predicted using in silico tools and confirmed by an in vitro functional assay.

The pedigree of the family included in this study was consistent with the presence of a de novo variant or with recessive autosomal or X‐linked inheritance, and filtering processes revealed the missense substitution c.916G>C in the *HS6ST2* gene as the unique candidate variant with high confidence. This variant was never been previously reported and encodes *HS6ST2,* which transfers sulphate from PAPS to the N‐sulphoglucosamine (GlcNS) residue in HS proteoglycans.

Severe myopia is arbitrarily defined as short sightedness greater than six diopters.^14^ It is usually an isolated disorder; however, more rarely, it can be associated with syndromic conditions (eg, Stickler syndrome).^15^ Recently, Li and Zhang reviewed 131 forms of syndromic myopia and reported that ID represents a variable feature in only a small fraction of them, including muscular dystrophy‐dystroglycanopathy, spastic paraplegia with psychomotor retardation, homocystinuria, retinal dystrophy (early onset), microphthalmia‐syndromic 6, and ichthyosis‐spastic quadriplegia‐mental retardation, along with the following syndromes: Cohen, Bohring‐Opitz, Pitt‐Hopkins, Poretti‐Boltshauser, Hamamy, Cornelia de Lange, and Danon. Of the 131 reported forms of syndromic myopia, 13 were X‐linked, and among them only Danon syndrome was sporadically associated with ID.^16^

HSPGs are ubiquitous components of the cell surface, extracellular matrix, and basement membranes, and interact with various ligands to influence cell growth, differentiation, adhesion, and migration.^13^ Multiple intercellular signaling molecules are regulated by HSPGs, including sonic hedgehog (SHH), bone morphogenetic protein (BMP), retinoic acid (RA), and fibroblast growth factor (FGF), along with Wingless/Integrated (WNT) signaling proteins, which are required for proper mammalian retinal development. Specifically, SHH controls the proximal‐distal patterning of the optic vesicle; BMP and RA regulate dorsal‐ventral differentiation; FGF signaling is required for the regionalization of the neural retina as well as for optic disc and fissure development; and WNT is necessary for the specification of the retinal pigmented epithelium. Low HSPG sulfation levels, as a result of mutations in 6‐O‐sulfotransferase genes, such as *HS6ST2*, are implicated in defective optic disc and stalk morphogenesis during mammalian visual system development.^13^

Altered *HS6ST2* expression has been identified in numerous human cancers,^17^ and is reported to participate in the pathogenesis of osteoarthritis and Kashin‐Beck disease.^18^ To date, there is no evidence of HS6ST2 involvement in Mendelian diseases.

The Face2Gene phenotyping tool identified a slight similarity of the phenotype of the affected boys with that of BWB syndrome, a condition described by Brooks et al., Morava et al., and Friez et al.^19^, ^20^, ^21^, ^22^ ID, speech delay, and severe myopia in a context of mild dysmorphia were the specific key features supporting the similarity. Moreover, the three BWB patients described by Morava et al.^21^ presented with increased blood lactate values in infancy, similar to the affected twins. Despite this, WES analysis excluded the presence of pathogenic variants of the *HUWE1* gene, which was identified by Friez et al. as a causal gene for BWB.^22^ In addition, there were clinical discrepancies between the affected probands and the BWB patients described in the literature. The boys described by Brooks et al.^19^ presented with spastic diplegia, optic and cerebellar atrophy, and entropion, which were not noted in our cases; the majority of patients reported by Morava^20^, ^21^ exhibited regression, seizures, and corpus callosum hypogenesis that were not present in the patients described in the current study. Given that BWB syndrome is an extremely rare disorder and exhibits a high degree of clinical heterogeneity,^19^, ^20^, ^21^, ^22^ it is possible that BWB could be genetically heterogeneous and that the phenotype of our patients represents a BWB‐like disorder, caused by mutation in a gene other than *HUWE1*. On the other hand, STRING network analysis of protein associations (https://string-db.org) confirmed that the E3 ubiquitin ligases *HUWE1* and *HS6ST2* do not share functional pathways, which does not support a BWB‐like etiology for our patients.

The c.916G>C variant is predicted to modify the 3D conformation of both isoforms of the HS6ST2 enzyme, since it is in a region common to both proteins. The mutation alters both the catalytic function of the enzyme and likely its localization in the membrane of the Golgi apparatus. The HS6ST2‐G306R functional assay demonstrated a consistent reduction in its 6‐O‐sulfotransferase function; however, since the assay was performed using total protein extracts and labeled HS substrate, no details are available about the possible membrane localization defect of the mutant isoform hypothesized to result from the misfolding of the N‐terminal residues.

HSPGs reside on the plasma membranes of all animal cells studied to date and are major components of the extracellular matrix. Studies of model organisms have demonstrated their importance in development and normal physiology.^23^ HS have been specifically noted in eye development, cranial axon guidance, and motor neuron migration.^24^, ^25^

In a mouse model, abnormally low sulfation of HS allows normal retinal neurogenesis and optic fissure closure; however, it leads to defective optic disc and stalk development.^24^ Adult mutant animals develop optic nerve aplasia/hypoplasia and exhibit retinal degeneration.^24^ Although myopia is not directly related to optic nerve aplasia/hypoplasia, but rather occurs if the eyeball is too long or the cornea is too curved, a link may exist between the mutation detected in the *HS6ST2* gene and the severe myopia observed in the affected children.

In conclusion, we identified a novel c.916G>C variant in the *HS6ST2* gene. The mutation caused a significantly reduction in HS6ST2 6‐O‐sulfotransferase activity and was associated with a previously undescribed form of syndromic XLID with severe congenital myopia. Although the phenotype of the probands in this study is weakly similar to BWB, the underlying genetic cause differs. A hypothesis could suggest possible loci and pathway heterogeneity in BWB syndrome. Alternatively, *HS6ST2* may cause a novel clinical condition and be pivotal for the development of myopia and cognitive impairment. Although the current report describes only a single pedigree, it could be used by clinicians to facilitate recognition of similar families and to provide a more accurate genetic counseling.

## CONFLICTS OF INTEREST

None of the authors have any conflicts of interest to declare.

## Supporting information


**Appendix S1:** autosomal recessive variants identified in the affected twins by whole exome sequencing analysis

**Appendix S2:** X‐linked recessive variants identified in the affected twins by whole exome sequencing analysis

**Appendix S3:** de‐novo variants identified in the affected twins by whole exome sequencing analysis
